# Patterned thin metal film for the lateral resolution measurement of photoacoustic tomography

**DOI:** 10.1186/1475-925X-11-37

**Published:** 2012-07-13

**Authors:** Do-Hyun Kim, Dong-Ho Shin, Sang Hun Ryu, Chul-Gyu Song

**Affiliations:** 1 Center for Devices and Radiological Health, U.S. Food and Drug Administration, 10903 New Hampshire Ave, Silver Spring, MD, 20993, USA; 2 Department of Electrical Engineering, Chonbuk National University, Jeonju, Jeonbuk, 561-756, South Korea

**Keywords:** Photoacoustic tomography, Resolution measurement, Ultrasound imaging.

## Abstract

**Background:**

Image quality assessment method of photoacoustic tomography has not been completely standardized yet. Due to the combined nature of photonic signal generation and ultrasonic signal transmission in biological tissue, neither optical nor ultrasonic traditional methods can be used without modification. An optical resolution measurement technique was investigated for its feasibility for resolution measurement of photoacoustic tomography.

**Methods:**

A patterned thin metal film deposited on silica glass provides high contrast in optical imaging due to high reflectivity from the metal film and high transmission from the glass. It provides high contrast when it is used for photoacoustic tomography because thin metal film can absorb pulsed laser energy. An US Air Force 1951 resolution target was used to generate patterned photoacoustic signal to measure the lateral resolution. Transducer with 2.25 MHz bandwidth and a sample submerged in water and gelatinous block were tested for lateral resolution measurement.

**Results:**

Photoacoustic signal generated from a thin metal film deposited on a glass can propagate along the surface or through the surrounding medium. First, a series of experiments with tilted sample confirmed that the measured photoacoustic signal is what is propagating through the medium. Lateral resolution of the photoacoustic tomography system was successfully measured for water and gelatinous block as media: 0.33 mm and 0.35 mm in water and gelatinous material, respectively, when 2.25 MHz transducer was used. Chicken embryo was tested for biomedical applications.

**Conclusions:**

A patterned thin metal film sample was tested for its feasibility of measuring lateral resolution of a photoacoustic tomography system. Lateral resolutions in water and gelatinous material were successfully measured using the proposed method. Measured resolutions agreed well with theoretical values.

## Background

Photoacoustic (opto-acoustic) imaging is an imaging modality which combines the physics of photonics (optics) and ultrasound (acoustics), providing high contrast of optical modality and long signal delivery path of ultrasound [1]. Sub-microsecond optical pulses generate surface or subsurface acoustic ultrasound waves when the pulses are irradiated on photon absorbing structure in biological samples. Generated acoustic waves are collected by an ultrasonic transducer, and then the image is reconstructed by computational procedure. Photoacoustic tomography (PAT) conventionally refers to a photoacoustic imaging technique by which expanded laser irradiation excites a large area of the sample while a moving transducer or multiple array of transducers collect acoustic waves [2]. In contrast to the photoacoustic microscopy (PAM) by which focused laser irradiation excites very small volume of acoustic wave and then is detected by a focused transducer [3], image reconstruction of PAT resembles that of ultrasonography. For PAM, traditional lateral resolution measurement method using a patterned resolution target such as the US Air Force (USAF) 1951 bar chart has been used [4].

One of the technical difficulties in PAT system development is the lack of a proper image target with which image quality parameters such as resolution can be adjusted and assessed. Tissue-mimicking phantom development for photoacoustic imaging system has recently been investigated [5,6], however, resolution targets are not widely adapted yet. There was an attempt to utilize photon-absorbing parallel lines printed on transparency for measurement of lateral resolution of a PAT system [7]. A resolution target for a PAT must contain photon-absorbing fine patterns which are embedded in the optically non-absorbing material. Various PAT image reconstruction algorithms have been developed, which in general rely on analytical solutions to the photoacoustic wave equation assuming the samples are acoustically homogeneous for simplicity [8]. However, difference in acoustic impedance of hetero-materials affects the quality of image reconstruction [9].

In this study, lateral resolution measurement of a PAT system using a patterned thin metal film will be discussed. Lateral direction in this study refers to the ultrasound propagation direction which is perpendicular to the light propagation. It must be noted that the lateral direction in PAT imaging is the axial direction of reflective ultrasonography.

## Methods

### Identifying ultrasound component

Laser-generated ultrasound in solid has been widely studied since the 1960’s. Different regimens for the production of ultrasound in metals using lasers with wavelengths in visible to near infrared range are summarized extensively in Davies’ review article [10]. Although the propagation of laser-generated ultrasound within the object where it was generated is well understood, its propagation in the surrounding media needs more study. The propagation of laser-generated ultrasound from thin metal film deposited on a base material immersed in water is especially of great interest in this study. Propagation of sound waves in the direction perpendicular to the surface of chromium film from the normal laser irradiation was extensively studied by Ko, *et al.*[11,12]. Propagation of sound waves generated in a 0.4-mm-thick stainless steel plate in water for transversal direction was observed by Schlieren imaging [13]. It was demonstrated that various types of sound wave can be formed by different generation mechanisms. The strongest ultrasound signal is generated from the surface vibration in the perpendicular direction to the surface, while weaker ultrasound signal still propagates along the surface mediated by the shear mode of ultrasound in metal [13].

In this study, we investigate a patterned thin metal film (PTMF) deposited on a transparent plate for its feasibility as a resolution test target for PAT. A commercially available chromium film deposited on a 2-mm fused silica glass with the USAF 1951 resolution bar-chart pattern was used. Positive patterns (Edmund Optics, NT57-896) and negative patterns (Edmund Optics, NT57-895) were used to compare the effect of phase of the generated ultrasound in propagating through a medium. These particular PTMFs are not optimal test targets for PAT because some of their patterns are only suitable for optical characterization. However, they were chosen in this study for their convenience of acquisition. Optimal patterns for PAT will be discussed in Section 4.

**Figure 1:**
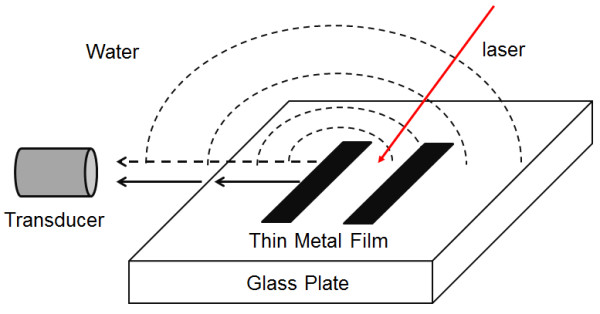
**Illustration of the sound propagation in water.** Ultrasound generated at the chromium strips propagates along the surface of the fused silica plate in the form of shear wave (solid arrow) and in the form of bulk wave (dashed arrow).

The major challenge in this work is to distinguish components of the ultrasound generated on the metal film delivered to the transducer for PAT image reconstruction. Details of the problem are illustrated in Figure 1.

The strongest component of laser-generated ultrasound from PTMF is the bulk wave propagating away from the PTMF which acts as the sound source [11,12]. However, shear wave (or surface acoustic wave) not only propagates within the fused silica plate, but it also generates sound waves in the water with the speed of the shear wave in the propagating direction [13]. Depending on the sound component detected by the transducer, measured resolution of PAT using PTMF will be either for the imaging in the medium (water) or in the silica. The spatial resolution (Δλ_ultrasound_) of a PAT system using a transducer with bandwidth (Δ*f*_transducer_) is determined by the relation:

(1) $$\Delta \lambda_{\mathrm{ultrasound}}=\frac{v_{\mathrm{ultrasound}}}{\Delta f_{\mathrm{transducer}}},$$

where v_ultrasound_ represents the speed of sound in the medium. For the PAT system, it is a commonly accepted fact that the lateral resolution of the system is half of Eq. (1) [14], [15]. The speeds of sound are the material property. Speed of sound in fused silica and in water are ~3,800 m/sec and ~1,500 m/sec, respectively. Since the speed of sound in fused silica is more than twice that in water, the resolution of a PAT system measuring the sound propagating in fused silica will be more than twice lower than that in water. Identifying the correct sound component is of great importance.

### Experiments

**Figure 2:**
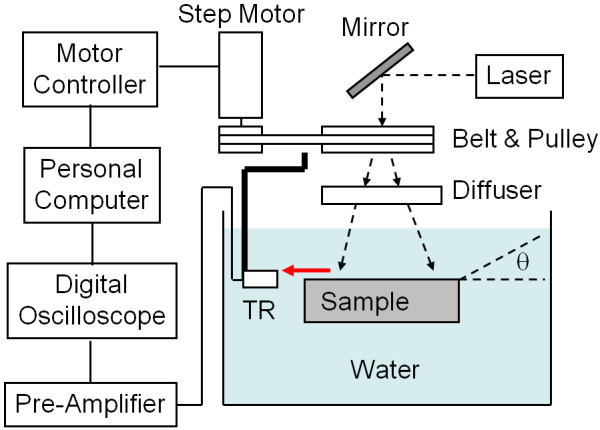
**Schematic of the PAT setup.** Solid black lines represent electrical connections, dashed lines represent laser paths, and red line represents ultrasound path. TR is the transducer. PTMF sample is placed such that the metal film is facing toward the incoming laser.

The schematic of the experimental setup is shown in Figure 2. The imaging system is based on an Nd:YAG laser (Meditech, Eraser-k) which has pulse energy of 400 mJ, center wavelength of 532 nm, and pulse duration of 8 nsec. A transducer (Panametrics-NDT, V323) having a bandwidth of 2.25 MHz was rotated around the sample using a step-motor connected to a belt-pulley assembly for a two-dimensional (2D) PAT image. 2D PAT imaging requires full rotation (360 degrees) of the transducer around the sample at 1.2 degrees per step, so 300 step-rotations. The ultrasonic signal collected by the transducer was amplified and then transferred to an oscilloscope (Tektronix, TDS2002), and then to a controlling personal computer via GPIB communication protocol. Image reconstruction was achieved using Labview (National Instrument) and Matlab (Mathworks) software. Uniform beam profile was ensured by choosing a proper diffuser.

## Results and discussion

### Measurement of lateral resolution in water

**Figure 3:**
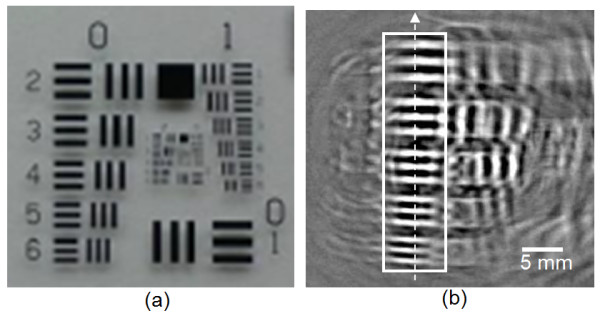
**(a) Digital photography and (b) a full 360 degree PAT image of the positive PTMF sample.** Patterns within the white rectangle represent Elements 2–6 of Group 0. Dashed-arrow represents the direction of 1D intensity profile measured by the transducer at a single fixed position.

Shown in Figure 3(a) is the positive USAF 1951 resolution target used as the PTMF sample. Figure 3(b) shows the 2D PAT image of the region in the proximity of Group 0, Elements 2 – 6. The PTMF sample and the transducer were immersed in water. As can be seen in Figure 3(b), all the Elements (2 – 6) of Group 0 are clearly distinguished. Other elements did not produce clean images either due to their small sizes beyond the system resolution limit or due to interference of multiple reflections from glass plate edges.

**Figure 4:**
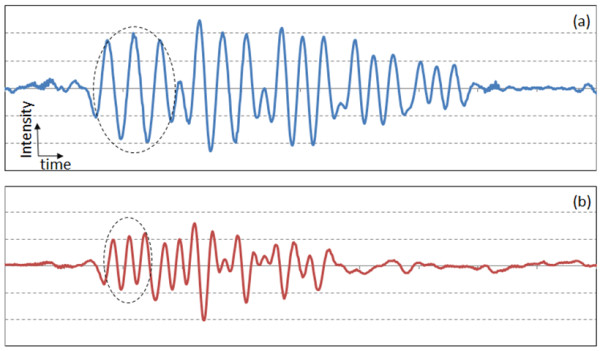
**Longitudinal 1D transducer signals from the positive PTMF sample: (a) Elements 2 – 6 of Group 0; (b) Elements 1 – 6 of Group 1.** The scales of x-axes of (a) and (b) are identical. Dashed circles indicate the signal from the largest element of each group.

For the measurement of lateral resolution, measurement of only one-dimensional (1D) distribution of signal amplitude along the longitudinal direction (dashed arrow in Figure 2) sufficiently provides the information of the lateral resolution of PAT system [14]. Compared to the 2D imaging, acquisition of only 1D data significantly reduces total measurement time. Figures 4(a) and 4(b) show the 1D signal from the positive PTMF sample placed on a transparent gelatinous block in water. Since the main purpose of this study is to distinguish the number of peaks from 1D intensity graphs, intensity and time scales are not specified in all 1D graphs. In this proof-of-concept study, a high energy laser (400 mJ) and non-scattering media were used to increase the contrast in the signal. Thus, variation between signal peaks was not significant enough to necessitate signal processing such as averaging. However, signals were averaged 5 times to reduce random noise from transducer and laser.

The speed of sound in water is ~1,500 m/sec, which gives the theoretical axial resolution of a transducer (2.25 MHz bandwidth) to be 0.33 mm. Signals from Elements 2 – 6 of Group 0 were shown in Figure 4(a). All the elements of Group 0 were distinguished. Element 2 of Group 0 has 1.12 line-pairs-per-mm (lpm), thus the distance between the peaks should be 0.89 mm. After converting the time scale of the data to the distance scale using the speed of sound, the measured distance between the two peaks from Element 2 of Group 0 was 0.90 mm, which is close to the actual value. For a PAT system with lateral resolution of 0.33 mm, three peaks from the element with 2.83 lpm (Element 4 of Group 1) should be distinguishable, while the peaks from the element with 3.17 lpm (Element 5 of Group 1) will start to merge with each other. Figure 4(b) shows the 1D signal from the Group 1 patterns of the same positive PTMF sample. As can be seen from the figure, three diminished peaks are observed in element 4, while only two peaks are observed in element 5. Thus, the PAT system resolution for water is between 0.32 mm and 0.35 mm. When the limited range of patterns is considered, the measured resolution measured from the PTMF is close to the theoretical resolution limit (0.33 mm).

**Figure 5:**
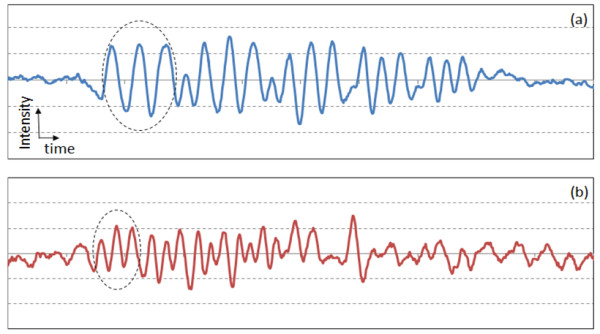
**Inverted 1D transducer signals from the negative PTMF sample: (a) Elements 2 – 6 of Group 0; (b) Elements 1 – 6 of Group 1.** The scales of x-axes of (a) and (b) are identical. Dashed circles indicate the signal from the largest elements of each group.

Figure 5(a) and 5(b) are for the 1D signal from the negative PTMF sample placed on a transparent gelatinous block. Signals were averaged 5 times. In comparison to the positive PTMF, in which chromium bars are deposited on a transparent fused silica plate, the surface of the negative PTMF sample is covered with thin chromium film while the bars (patterns) are left uncovered. The words positive and negative are adaptation from the traditional photography film. Negative PTMF were tested for comparison because it has wider coverage of chromium film from which photoacoustic signal is generated. After signal acquisition, 1D signal was inverted in order for direct comparison with that from positive PTMF. Compared to the results shown in Figure 4, the results from the negative PTMF shown in Figure 5 revealed higher signal-to-noise ratio due to increased signal level from larger chromium area. All the peaks of Group 0 elements were clearly distinguished in Figure 5(a). For Group 1, three diminished peaks are observed in element 4, while only two peaks are observed in element 5, as can be seen in Figure 5(b). Thus, the PAT system resolution for water is between 0.32 mm and 0.35 mm. The measurement result is basically the same as that from positive PTMF.

### Identifying the sound component

In Section 3.1, the lateral resolution of the PAT system was measured using PTMF samples based on the assumption that the laser-generated ultrasound only propagates in the surrounding water. According to Don-Liyanage’s study [13], shear wave generated by the surface acoustic wave in solid propagates in all directions. However, compared to the bulk wave which propagates with the same speed in all directions, shear wave has higher speed in the direction parallel to the surface. Thus, if the detected signal is from shear wave, the measured resolution will depend on the angle between the transducer and the surface. If no dependency is observed, then the detected signal is bulk wave.

To identify which component of the sound was detected in this experiment, PTMF sample was tilted by the amount of angle θ (see Figure 2) such that the patterned metal film faced toward the transducer. The transducer axis was still facing toward the center of the PTMF sample. The lateral distance between the bars of the pattern will decrease by the amount of cos(θ). For example, if the tilting angles were θ = 30 degrees and θ = 45 degrees, the minimal spacing of bar patterns that can be distinguished by the PAT system will be 0.38 mm (= 0.33/cos30) and 0.47 mm (= 0.33/cos45), respectively. The minimum spacing resolvable by Elements 3 and 4 of Group 1 ranges from 0.35 mm to 0.40 mm, thus Element 3 or 4 of Group 1 will be able to be distinguished for a 30-degree tilted PTMF sample. The minimum spacing resolvable by Elements 1 and 2 of Group 1 ranges from 0.47 mm to 0.50 mm, thus Element 1 or 2 of Group 1 will be able to be distinguished for a 45-degree tilted PTMF sample.

**Figure 6:**
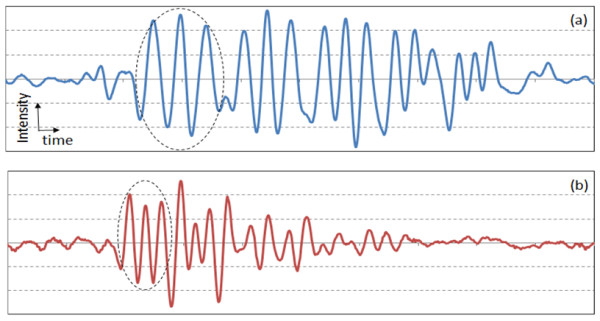
**Inverted 1D transducer signals from the negative PTMF sample tilted 30 degrees toward the transducer: (a) Elements 2 – 6 of Group 0; (b) Elements 1 – 6 of Group 1.** The scales of x-axes of (a) and (b) are identical. Dashed circles indicate the signal from the largest elements of each group.

Figure 6(a) and 6(b) show the 1D signal from the negative PTMF sample tilted 30 degrees toward the transducer. Signals were averaged 5 times. All the elements of Group 0 were distinguished as can be seen in Figure 6(a). Elements 1 and 2 of Group 1 were clearly distinguished as can be seen from Figure 6(b). A previous prediction was that the peaks will collapse between Elements 3 and 4 of Group 1, however, peaks of Element 3 of Group 1 were observed to be collapsed. The spacing between bars of Element 2 and 3 of Group 1 ranges from 0.40 mm to 0.45 mm, which gives the range of the tilt-angle (θ) from 34 degrees to 42 degrees. The error range is broad, which is the disadvantage of using a readily available USAF 1951 resolution target for PAT measurement. Measurement error in θ may have been introduced from the measurement of angle from outside of the water tank. The actual angle between the surface of the PTMF sample and the transducer axis should be 35 degrees rather than 30 degrees.

**Figure 7:**
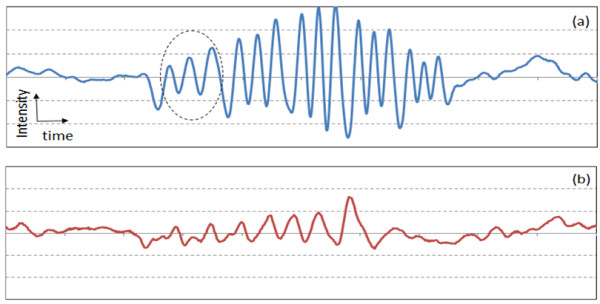
**Inverted 1D transducer signals from the negative PTMF sample tilted 45 degrees toward the transducer: (a) Elements 2 – 6 of Group 0; (b) Elements 1 – 6 of Group 1.** The scales of x-axes of (a) and (b) are identical. Dashed circles indicate the signal from the largest element of each group.

Figure 7(a) and 7(b) show the 1D signal from the negative PTMF sample tilted 45 degrees toward the transducer. Signals were averaged 5 times. Contrary to the prediction that up to Element 1 or 2 of Group 1 will be distinguished, only the elements of Group 0 were distinguished. Adapting the 5 degrees of error assumed from the previous results for 30-degree tilt, the actual tilt angle is assumed to be 50 degrees rather than 45 degrees. For θ = 50 degrees, the spacing between the bars that leads to 0.33 mm lateral spacing is 0.51 mm, which lies between Element 1 of Group 1 and Element 6 of Group 0. Figure 7(b) shows that none of the peaks from the Group 1 patterns was distinguished, thus the assumption of 5-degree measurement error is still valid.

From the two validation experiments with tilted samples, it is concluded that the ultrasound signals measured in this study demonstrated the same speed regardless of the location of transducer relative to the sample surface where they were measured. It is proven that the observed ultrasound was bulk wave. Intensities of ultrasound signals did not dramatically vary for different transducer locations (sample tilt angles), which is another proof that the observed ultrasound was not shear wave. Spherical bulk wave is the mode of propagating shock wave in this study. The resolution measured by the proposed method is the resolution in the media in which the sample is immersed, rather than the resolution in the sample block.

### Practical applications

A practical design of the resolution target for PAT system is discussed. Although it was proven in this study that the patterned thin metal film deposited on a glass plate can be effectively utilized for measuring the lateral resolution of PAT system in a medium, commercially available USAF 1951 target needs modification for practical reasons. First, small patterns designed for optical resolution measurements induce unnecessary interferences as was shown in Figure 3(b). Second, although the speed of sound in biological tissue (for example in liver 1570 m/sec) [16] is close to that in water (1480 m/sec at room temperature) [17], there still is discrepancy between two values. A practically useful resolution target for PAT could be made by a PTMF plate having only a limited number of patterns embedded in a transparent material (or tissue-mimicking scattering material) block which has a speed of sound similar to that of biological tissue. The speed of sound in gel material is 1580 m/sec [16]. Resolution of the PAT system for gelatinous material is close to that for some biological tissues.

**Figure 8:**
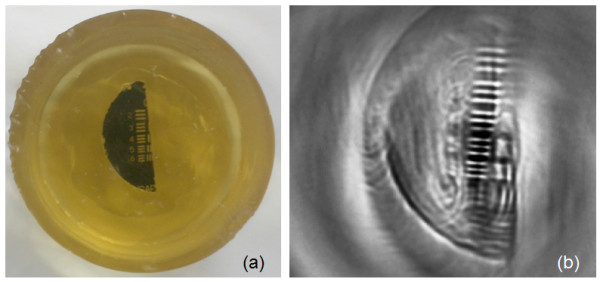
**(a) Digital photography of the suggested resolution target. A piece of PTMF sample with only Group 0 patterns was embedded in a gelatinous block; (b) 2D reconstruction of PAT image from the suggested resolution target.** Inverted signal was used for this particular resolution target made of negative PTMF sample.

Figure 8(a) shows the digital photography of the suggested resolution target. Only one group of patterns from the negative PTMF sample was cut into a piece so that interference from unresolvable smaller patterns can could be prevented. To reflect the resolution of the PAT system for a biological tissue, the PTMF piece was embedded in a gelatinous block in which the speed of sound is similar to that of tissues. Figure 8(b) shows the 2D PAT image from the suggested resolution target. Image reconstruction process was the same as that used in obtaining Figure 3(b). However, the signals were inverted because of the negative pattern on the PTMF sample. Although some unnecessary patterns were still remaining due to the error in cuts, Figure 8(b) shows that the acquired 2D PAT image for the Group 0 patterns is clean enough to distinguish all 5 elements. For actual preparation of such resolution targets, line pairs with more variety of spatial frequencies may be used.

**Figure 9:**
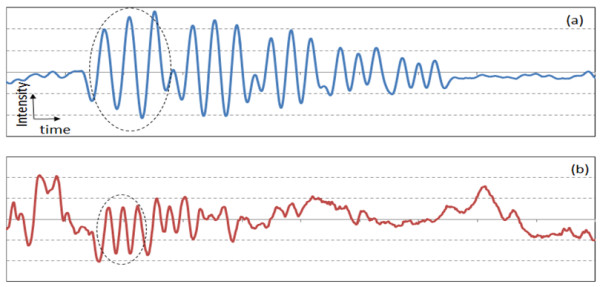
**Inverted 1D transducer signals from the negative PTMF sample embedded in a gelatinous block.** (**a**) Elements 2 – 6 of Group 0; (**b**) Elements 1 –6 of Group 1. The scales of x-axes of (a) and (b) are identical. Dashed circles indicate the signal from the largest element of each group.

Figure 9a) and 9(b) show the 1D signal from the suggested resolution target. Signals were averaged 5 times. All the elements of Group 0 were distinguished as can be seen in Figure 9(a). Element 2 of Group 1 showed three distinctive peaks in Figure 9(b), while Element 3 of Group 1 showed slightly diminished peaks. Peaks started to collapse for the Element 4 of Group 1 in water as was shown in Figure 5(b). This is due to the fact that the lateral resolution of the PAT system for gelatinous material is 0.35 mm, which is slightly poorer than that for water (0.33 mm). The results coincide well with theoretical prediction.

**Figure 10:**
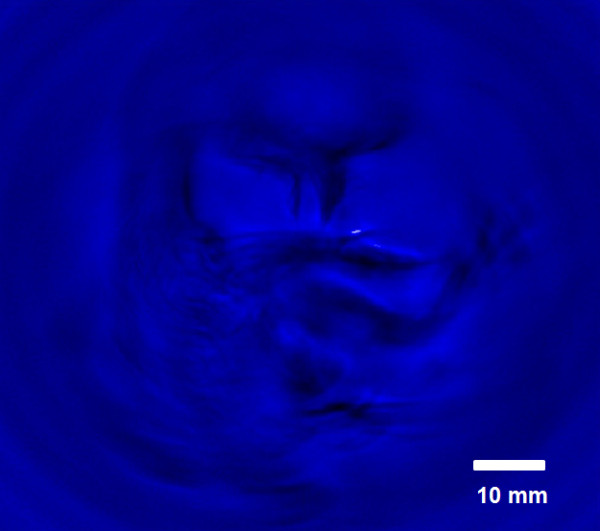
**Chicken embryo of day 12 imaged by the developed PAT system.** The sample was submersed in water bath.

The developed PAT system was fully characterized for the lateral resolution. It exhibited the resolution that agrees well with the theoretical value. For future work, the PAT system was tested for biomedical application. Figure 10 shows a PAT image of chicken embryo on day 12. The embryo was extracted from the egg shell, and was placed on a transparent gelatinous block. The whole sample was submersed in water during the image reconstruction procedure. The transducer had bandwidth of 2.25 MHz. At this stage, chicken embryo starts to develop feathers in the tail. Developing toes reaches a thickness close to 1.0 mm. These fine structures can only be identified by an imaging system with proper resolution. The current PAT system with the measured lateral resolution of 0.33 mm in water demonstrated the required resolving power as can be seen in Figure 10.

## Conclusions

Patterned thin metal film deposited on fused silica plate was investigated for the measurement of lateral resolution of a PAT system. Propagation of bulk ultrasound wave was confirmed by this proof-of-concept experiment. Lateral resolutions measured by this method for different materials agreed well with theoretical limit. The lateral resolution measured by this method is the resolution of the PAT system in the media rather than the resolution in the sample material. A practically useful resolution target block was suggested and tested. To the best of the authors' knowledge, this finding is the first systematic experimental confirmation using a resolution target showing that the lateral resolution of a PAT system (with a fixed bandwidth of the transducer) is the half of the theoretical spatial resolution expressed by Eq. (1). Although controlled experimental results presented in this study using high power laser and non-scattering media successfully proved the concept, for this method to be fully standardized and validated for biological samples or tissue simulating phantoms, a quantitative discrimination method of signal peaks will be studied and suggested in future works.

## Competing interests

The authors declare that they have no competing interests.

## Authors' contributions

DHK proposed the idea, analysed the data, and composed the manuscript. DHS and SHR performed experiments. CGS supervised and provided support to DHS and SHR. All authors read and approved the final manuscript.
